# Nuclear-Encoded Plastidal Carbonic Anhydrase Is Involved in Replication of *Bamboo mosaic virus* RNA in *Nicotiana benthamiana*

**DOI:** 10.3389/fmicb.2017.02046

**Published:** 2017-10-18

**Authors:** I.-Hsuan Chen, April Y. Tsai, Ying-Ping Huang, I.-Fan Wu, Shun-Fang Cheng, Yau-Heiu Hsu, Ching-Hsiu Tsai

**Affiliations:** ^1^Graduate Institute of Biotechnology, National Chung Hsing University, Taichung, Taiwan; ^2^Research Center for Sustainable Energy and Nanotechnology, National Chung Hsing University, Taichung, Taiwan

**Keywords:** carbonic anhydrase, *Bamboo mosaic virus*, *Nicotiana benthamiana*, RNA replication, *in vitro* replication, initiation/elongation switch

## Abstract

On inoculation of *Nicotiana benthamiana* with *Bamboo mosaic virus* (BaMV), a gene with downregulated expression was found involved in the infection cycle of BaMV. To uncover how this downregulated gene affects the accumulation of BaMV in plants, we used loss- and gain-of-function experiments. Knockdown of this gene decreased the accumulation of BaMV coat protein to approximately 60% in both plants and protoplasts of *N. benthamiana* but had no effect on *Potato virus X* and *Cucumber mosaic virus* infection. The full-length gene was cloned and revealed as an *N. benthamiana* nuclear-encoded chloroplast carbonic anhydrase (CA) and so designated *NbCA*. As compared with the accumulation of BaMV RNAs in *NbCA*-knockdown protoplasts, both plus- and minus-strand RNAs were reduced. We further fused *NbCA* with Orange fluorescent protein to confirm its localization in chloroplasts on confocal microscopy. However, transiently expressed NbCA in chloroplasts did not considerably increase BaMV accumulation. The addition of exogenous CA may not have any additive effect on BaMV accumulation because of the natural abundance of CA in chloroplasts. In an *in vitro* replication assay, the addition of *Escherichia coli*-expressed NbCA enhanced exogenous template level (re-initiation and elongation) but not endogenous template level (only elongation). These results suggest that NbCA is possibly involved in re-initiation step of BaMV RNA replication. Further analysis indicated that proton concentration could influence the endogenous and exogenous template activities. Hence, our results implied that NbCA could be playing a role in harnessing proton concentration and favoring the replicase with the re-initiation template.

## Introduction

*Bamboo mosaic virus* (BaMV), belonging to the *Potexvirus* genus of family *Alphaflexiviridae* (Lin et al., 1994), has one single-stranded positive-sense RNA genome of approximately 6.4 kb long [excluding the poly(A) tail]. The genome comprises a 5′ cap structure, 3′ poly(A) tail, and five open reading frames (ORFs 1-5) (Lin et al., 1994). ORF1, encoding a 155-kDa polypeptide, harbors three functional domains (Meng and Lee, 2017): the capping enzyme domain, which exerts AdoMet-dependent guanylyltransferase activity (Li et al., 2001a; Huang et al., 2004; Hu et al., 2011); the helicase-like domain, which contains NTPase and RNA 5′-triphosphatase activities (Li et al., 2001b); and the RNA-dependent RNA polymerase (RdRp) core domain (Li et al., 1998; Cheng et al., 2001). ORFs 2-4, encoding 28-, 13-, and 6-kDa polypeptides, respectively, overlap and are called triple-gene-block (TGB), designated TGBp1, -2, and -3, respectively. The movement of BaMV requires these three TGB proteins (Lin et al., 2004, 2006; Chou et al., 2013). ORF5, encoding a 25-kDa polypeptide viral capsid protein (CP) is required for cell-to-cell movement, symptom development, and virion assembly (Lan et al., 2010; Hung et al., 2014a,b). The 3′ untranslated region (UTR) plays roles in minus-strand RNA initiation, polyadenylation, and long-distance movement (Chen et al., 2017).

Although bamboo is the natural host for BaMV, *Nicotiana benthamiana* is the major assay host for studying the infection cycle of BaMV at the molecular level. A putative methyltransferase was identified to play a role in restricting the accumulation of BaMV in a dose-dependent manner in protoplasts (Cheng et al., 2009). Glyceraldehyde 3-phosphate dehydrogenase was found to play an inhibiting role in regulating minus-strand RNA synthesis by binding to the 3′ UTR of BaMV RNA (Prasanth et al., 2011). The chloroplast phosphoglycerol kinase (PGK) interacts with the 3′ UTR, including part of the poly(A) tail, and ushers the viral RNA into the chloroplast for BaMV replication (Lin et al., 2007; Cheng et al., 2013a). A heat shock protein 90 homolog binds to the viral replicase, and the 3′ UTR enhances the early stage of BaMV replication (Huang et al., 2012). A glutathione transferase, NbGSTU4, interacts with the 3′ UTR of BaMV RNA and enhances the minus-strand RNA synthesis (Chen et al., 2013). Another viral replicase-associated host protein, XRN4, with RNase activity, assists the accumulation of BaMV (Lee et al., 2015). NbRabG3f, an Rab-GTPase protein, is involved in positive regulation of BaMV replication (Huang et al., 2016). The host factor Ser/Thr kinase-like protein (NbSTKL), localized mainly on the cell membrane, can facilitate BaMV intercellular movement (Cheng et al., 2013b). An RabGTPase-activating protein (NbRabGAP1) is involved in BaMV cell-to-cell and systemic movement (Huang et al., 2013).

Carbonic anhydrase (CA) is a zinc metalloenzyme that can catalyze the interconversion of carbon dioxide (CO_2_) and bicarbonate (HCO_3_^-^). The reaction of CO_2_ + H_2_O ↔ HCO_3_^-^ + H^+^ reaches equilibrium spontaneously but slowly and can be accelerated by the catalyzation of CA (Dimario et al., 2017). CA also plays vital roles in many biochemical processes that involve pH homeostasis and ion transport (Tashian, 1989) and carboxylation or decarboxylation reactions such as photosynthesis and respiration, respectively (Moroney et al., 2001). From the structures and amino acid sequences, CAs can be divided into five distinct classes: α, β, γ, δ, and ε, which share little sequence similarity and are assumed to have evolved independently (Hewett-Emmett and Tashian, 1996; Tripp et al., 2001; So et al., 2004; Sawaya et al., 2006; Floryszak-Wieczorek and Arasimowicz-Jelonek, 2017). The CAs of algae and plants are all belong to α, β, and γ classes, with the β class most prevalent (Moroney et al., 2001). Furthermore, in C3 plants such as *N. benthamiana*, CA is found in the stroma of mesophyll chloroplasts and has been found with some characteristics such as the ability to bind salicylic acid (SA), antioxidant activities in response to pathogens (Slaymaker et al., 2002; Restrepo et al., 2005), the provision of HCO_3_^-^ for lipid biosynthesis (Hoang and Chapman, 2002) and the regulation of CO_2_-mediated stomatal closure (Hu et al., 2010).

The relation between CA and plant pathogens has been studied lately. CA is identified as a SA-binding protein 3 (SABP3) and exhibits CA enzymatic, SA-binding, and antioxidant activities in *N. tabacum* plants. Furthermore, reducing the expression of CA in plants suppressed the hypersensitive reaction (HR) in disease resistance (Slaymaker et al., 2002). In CA-silenced *N. benthamiana* plants, the growth of *Phytophthora infestans* was considerably increased, probably also due to suppression of the HR (Restrepo et al., 2005).

Although this earlier research mostly documented that the host CA is necessary for plant defense, in this study, we found that CA could, by contrast, help BaMV accumulation. Therefore, we investigated how CA could play a role in assisting BaMV accumulation in plants. Furthermore, we studied whether CA is involved in the initiation step of BaMV replication in *N. benthamiana*.

## Materials and Methods

### Plants and Viruses

*Nicotiana benthamiana* plants were grown in a growth room at 28°C with 16 h light and 8 h dark. Three viruses were used for inoculation: BaMV strain S (Lin and Hsu, 1994), *Potato virus X* (PVX) strain Taiwan, and *Cucumber mosaic virus* (CMV) strain NT9 (Hsu et al., 1995).

### Virus-Induced Gene Silencing (VIGS) and Mechanical Inoculation of Viruses

The cDNA fragment of *ACAC10-1* (fragment of *NbCA* gene) was cloned into the pGEM-T Easy vector (Promega, Madison, WI, United States) in a previous study (Cheng et al., 2010). To use *ACAC10-1* in *Tobacco rattle virus* (TRV)-based VIGS (Liu et al., 2002) in *N. benthamiana* plants, *ACAC10-1* in the pGEM-T Easy vector was digested with *Eco*RI and subcloned into the pTRV2 vector to generate pTRV2-NbCA and transformed into *Agrobacterium* C58C1 strain. Furthermore, *Agrobacterium* carrying a pTRV2-containing luciferase (*Luc*) gene or phytoene desaturase (PDS) gene were used as a negative or positive control, respectively. *Agrobacterium* containing pTRV1, pTRV2-NbCA, pTRV2-Luc, or pTRV2-PDS was cultured at 30°C to OD_600_ 0.8∼1.0; the cells were collected by centrifugation at 5000 rpm, then suspended in the induction medium (10 mM MgCl_2,_ 10 mM MES pH5.6, and 150 μM acetosyringone) at 30°C for 1 h. After induction, equal volumes of both cultures (pTRV1 and pTRV2-NbCA, pTRV2-Luc or pTRV2-PDS) were mixed before agroinfiltration onto the second, third, and fourth leaf of true leaves of a 1-month old *N. benthamiana.* When the PDS-knockdown plants had a photo-bleach phenotype, 500 ng of BaMV virion was mechanically inoculated onto the fourth leaf above the infiltrated leaves.

The knockdown efficiency of *NbCA* was calculated by measuring the expression ratio normalized to the expression of *actin* between the *NbCA-*knockdown and control plants. Two sets of primers were used for the RT-PCR to amplify *NbCA* and *actin* gene expression: NbCA/F (5′-AGTGCATGTGGAGGTATCAAAGGT-3′)/NbCA/R (5′-GTCGACTACGGAAAGAGAAGG-3′) and actin/3′ (5′- GTGGTTTCATGAATGCCAGCA-3′)/actin/5′ (5′-GATGAAGATACTCACAGAAAGA-3′).

### Protoplast Preparation and Viral RNA Inoculation

The preparation of protoplasts from *N. benthamiana* and viral RNA inoculation was described previously (Tsai et al., 1999). Approximately 2 g of agroinfiltrated leaf was collected from the knockdown *N. benthamiana* and digested with pectinase and cellulase at 25°C overnight. The mesophyll protoplasts were isolated from the interface zone between the Mannitol-MES buffer and the sucrose. After a few washes, protoplasts were stained with fluorescein diacetate to examine the quality of cells under a fluorescent microscope. Approximately 2.5 × 10^5^ protoplasts were inoculated with 1.5 μg BaMV, PVX, or CMV viral RNA with 40% polyethyleneglycol-6000. Total protein or RNA was extracted from protoplasts and detected by western blot analysis or real-time qRT-PCR, respectively.

### Western Blot Analysis

The total protein of inoculated leaves or protoplasts was extracted with plant extraction buffer (50 mM Tris-HCl pH 6.8, 10% glycerol, and 2% SDS), boiled with the SDS sample buffer (10% glycerol, 12.5 μg/ml bromophenol blue, 10 mg/ml SDS, 125 mM Tris-HCl pH 6.8, 2.5% β-mercaptoethanol) for 5 min, separated on a 12% polyacrylamide gel containing 0.1% SDS, transferred onto a nitrocellulose membrane (PROTRAN BA 85 Schleicher and Schnell), and probed with primary antibody [anti-Orange fluorescent protein (OFP), -BaMV, -PVX, or -CMV] and with the secondary antibody [affinity purified anti-rabbit IgG conjugated IRDye 800 (ROCKLAND)]. Finally, membranes with fluorescent bands were scanned by using LI-COR Odyssey (LI-COR Biosciences). In addition, rbcL (RuBisCo large subunit) stained with Coomassie blue was used as a loading control.

### Total RNA Extraction

Total RNA was extracted from leaves with STE buffer (400 mM Tris-HCl pH8.0, 400 mM NaCl, and 40 mM EDTA), 1% SDS, and 3.3 mg/ml bentonite and an equal volume of phenol/chloroform. After ethanol precipitation, the RNA was further precipitated with 3 M NH_4_OAc, washed, dried, and dissolved in 30 μl de-ionized H_2_O. For RNA extracted from protoplasts, cells were mixed with 200 μl protoplast RNA extraction buffer (100 mM Tris-HCl pH 8.0, 10 mM EDTA, 100 mM NaCl, 1% SDS, and 600 μg bentonite). After phenol/chloroform extraction and ethanol precipitation, RNA was further precipitated with 3 M NH_4_OAc and dissolved in 13 μl de-ionized H_2_O.

### qRT-PCR

qRT-PCR was used to detect both BaMV plus- and minus-strand genomic RNA. The cDNA synthesis reaction involved use of ImProm-II Reverse Transcriptase (Promega, Carlsbad, CA, United States) as instructed with the primers for Oligo dT(25T) and BaMV+51 (5′-ACTGCCAATTGTCCCCTACA-3′) for the plus- and minus-strand, respectively. For quantifying the accumulation of BaMV genomic RNA or minus-strand RNA, primers for BaMV+51 and BaMV-282 (5′-TGTGCTGAACGGGTTATGAG-3′) or BaMV+1766 (5′-CACATCCGGCACTTACCA-3′) and BaMV-2002 (5′-ATGTATCACGGAAATAAGAGTT-3′) were used, respectively, in the reaction containing a 1000X dilution of SYBR green I (Cambrex Bio Science Rockland, ME, United States). qPCR was performed in 0.2-ml PCR tubes with 0.6 mM primer, 0.2 mM each deoxyribonucleoside triphosphate, 10 mM Tris-HCl pH 8.8, 1.5 mM MgCl_2_, 50 mM KCl, 0.1% Triton X-100, 2 μl cDNA, 3 units of Taq DNA polymerase (Promega) and RNase-free water to a final volume of 20 μl. Cycling conditions began with an initial hold at 95°C for 5 min, followed by about 30 cycles of 94°C for 30 s, 56°C for 30 s and 72°C for 30 s. Reactions were carried out in a RotorGene 3000 (Corbett Research, Sydney, Australia) with data acquisition at 72°C on the channel, excitation at 470 nm and detection at 585 nm, by using a high-pass filter for both plus- and minus-strand. The reaction without template or reverse transcriptase was a negative control, and *actin* was detected for normalization. All samples were run at least three times.

### NbCA Cloning and Visualizing Its Localization

The full-length CA cDNA of *N. benthamiana* was cloned into the pEpyon binary vector that carries the *mOrange2* reporter gene (Shaner et al., 2008) to express the fusion protein NbCA-OFP. The ORF of the *NbCA* was amplified with NbCA/F, 5′-GGATCCATGTCAACTGCTTCCA-3′, and NbCA/R, 5′-GGTACCTACGGAAAGAGAAG-3′ (*Bam*HI and *Kpn*I underlined, respectively). The PCR product was first cloned into the pGEM-T easy vector (Promega, Madison, WI, United States), then sub-cloned into pEpyon with *Bam*HI and *Kpn*I after sequence verification.

*Agrobacterium* containing the binary vector with NbCA-OFP or vector alone was cultured and infiltrated into *N. benthamiana* plants. The fluorescent signals were detected at 3 days post-infiltration by confocal laser scanning microscope (FV1000, Olympus). To observe whether NbCA altered its localization after BaMV infection, pKBG, a plasmid containing an infectious cDNA of BaMV with a GFP reporter (Prasanth et al., 2011) was co-infiltrated with NbCA-OFP. The fluorescent signals were detected at 4 days post-infiltration by confocal laser scanning microscope.

### Transient Expression of NbCA-OFP Fusion Protein

NbCA-OFP was transiently expressed via agroinfiltration on a leaf for 1 day and the BaMV virion was inoculated on the same leaf for another 3 days. The expression of this fusion protein and accumulation of BaMV coat protein were detected by western blot analysis.

### NbCA Expression and Purification from *Escherichia coli*

The coding sequence of *NbCA* without the predicted transit peptide (Slaymaker et al., 2002) was amplified with the primer set CAgene/F, 5′-GGATCCGAATTGCAATCATCAGATGG-3′, and CAgene/R, 5′-GCTCGAGTACGGAAAGAGAAGGAGAAA-3′ (*Bam*HI and *Xho*I site underlined, respectively). The PCR product was cloned into the pGEM-T easy vector and the sequence was verified. Finally, *NbCA* was subcloned from the T-vector into the pET29a(+) expression vector (Invitrogen) and transformed into *E. coli* BL21(DE3). The resulting clone was designated pET29a(+)-NbCA.

*Escherichia coli* containing pET29a(+)-NbCA was cultured to OD_600_ = 0.7 ∼ 1.2 (100 ml in total volume), the expression was induced with 1 mM isopropyl β-D-1-thiogalactopyranoside (IPTG) at 16°C for 1 day, then samples were centrifuged at 7000 rpm at 4°C for 7 min. The cell pellet was resuspended with 8 ml buffer A (50 mM NaH_2_PO_4_ pH 8.0, 300 mM NaCl) containing protease inhibitor cocktail (Roche, Germany) and subjected to the French Press to break cells: the sample was loaded into the French Press and squeezed out four times, then centrifuged at 12000 rpm at 4°C for 10 min. The supernatant was incubated with 1 ml complete His-tag Purification Resin (Roche, Germany) overnight, washed with 10 ml buffer A containing 50 mM imidazole, and eluted with buffer A containing 250 mM imidazole. Finally, the eluted protein was dialyzed with 150 ml buffer A four times to remove imidazole and stored at -80°C with the addition of final 10% glycerol. The vector-only construct was manipulated under the same condition as the negative control.

### Replicase Complex Preparation and *in Vitro* Replication Assay

*Bamboo mosaic virus*-infected leaves were collected at 5 dpi and homogenized with polytron in replicase complex extraction buffer (50 mM Tris-HCl pH 7.6, 15 mM MgCl2, 120 mM KCl, 0.1% β-mercaptoethanol, 20% glycerol, 1 μM pepstatin A, 0.1 mM phenylmethylsulfonyl fluoride) with a 2 ml/g of buffer/leaf ratio. The leaf slur was filtrated through Miracloth (Calbiochem) and centrifuged at 500 × *g* for 10 min to remove the cell debris. The pellet was resuspended in suspension buffer (50 mM Tris-HCl pH 8.2, 10 mM MgCl2, 1 mM dithiothreitol, 1 μM pepstatin A, 1 μM leupeptin) after centrifugation at 30,000 × *g* for 35 min. Approximately 2 ml of the extract was loaded on 28 ml of 20 ∼ 60% continuous gradient of sucrose with the gradient buffer (50 mM Tris-HCl pH 8.0, 10 mM NaCl, 1 mM EDTA, 5% glycerol, 1 μM pepstatin A, 0.1 mM PMSF, 1 mM dithiothreitol) and centrifuged at 72,100 × *g* for 7.2 h. The 5th and 6th fractions of the 10 fractions (from top to bottom) with the highest RdRp activity were pooled and stirred with 1.5% NP-40 for 1 h to solubilize the membrane-associated RdRp.

For the *in vitro* replication assay with the endogenous RNA templates, 15 μl of the replicase complex preparation (pH 8.0) was added to a total 50 μl reaction containing 2 mM (A, C, and G) TP, 2 μM UTP, 3 mM MgCl2, 10 mM dithiothreitol, 50 mM Tris-HCl pH 8.2 (6.8, 7.4, 7.8, 8.8, or 9.0 was used in testing the proton concentration for the *in vitro* replication assays), 12 unit RNase OUT (Invitrogen, Carlsbad, CA, United States), 8 mg bentonite, 0.066 μM [α-^32^P]UTP (3000 Ci mmol/1, Dupont-NEN) and 5.2 μg recombinant NbCA at 30°C for 1 h (the reaction made up of final pH is 8.1 when reaction buffer is 8.2). Therefore, the Tris-HCl buffer at different pH was used in the reaction to reach the final target pH as 7.1, 7.5, 7.8, 8.6, or 8.8, respectively. The reaction was stopped by adding 150 μl 5 mM EDTA, extracted with phenol/chloroform, and precipitated with ethanol. The radioactive RNA products were resolved on a 1% agarose gel and quantified by using the PhosphoImaging analyzer BAS-2500 (FUJIFILM).

For the exogenous RNA templates, 15 μl of the replicase complex preparation was first treated with 10 units of micrococcal ribonuclease containing 2.5 mM Ca(OAc)2 to remove endogenous RNA at 30°C for 30 min in a total volume of 11.5 μl reaction. Then the reaction was terminated by adding 16 mM EGTA and set on ice for 1 ∼ 2 min. An aliquot of 15 μl mixture was subjected to a total 50 μl reaction as in the endogenous RNA template reaction. The radioactive RNA products were resolved on a 5% polyacrylamide gel and quantified by using the PhosphoImaging analyzer BAS-2500 (FUJIFILM).

### RNA Preparation

Ba-77 RNA (the 3′-end 77 nt of BaMV minus-strand RNA) and r138/40A RNA (the 3′ UTR of BaMV RNA) was prepared in an *in vitro* transcription with plasmids constructed previously (Cheng et al., 2001). The reaction was carried out in 100 μl containing 10 μg linearized plasmid (*Eco*RI and *Bam*HI for Ba-77 and r138/40A, respectively, in pUC18), 40 mM Tris-HCl pH 8.0, 2 mM spermidine, 8 mM MgCl_2_, 10 mM dithiothreitol, 0.4 mM NTP and 200 U T7 RNA polymerase at 37°C for 2 h. The RNA was then gel purified, quantified, and stored at -80°C.

## Results

### The Accumulation of BaMV in NbCA-Knockdown Plants Is Reduced

The sequence of a gene, *ACAC10-1*, found downregulated in *N. benthamiana* plants by cDNA-amplified fragment polymorphism (AFLP) after BaMV inoculation (Cheng et al., 2010), showed 100% match with an EST clone 30F62 containing a nuclear-encoded chloroplast CA gene. The gene was designated *NbCA*. To gain a better understanding of the relation between *NbCA* and BaMV infection, we inoculated BaMV virion into leaves of *N. benthamiana* with *Tobacco rattle virus* (TRV)-based *NbCA* knockdown.

**FIGURE 1:**
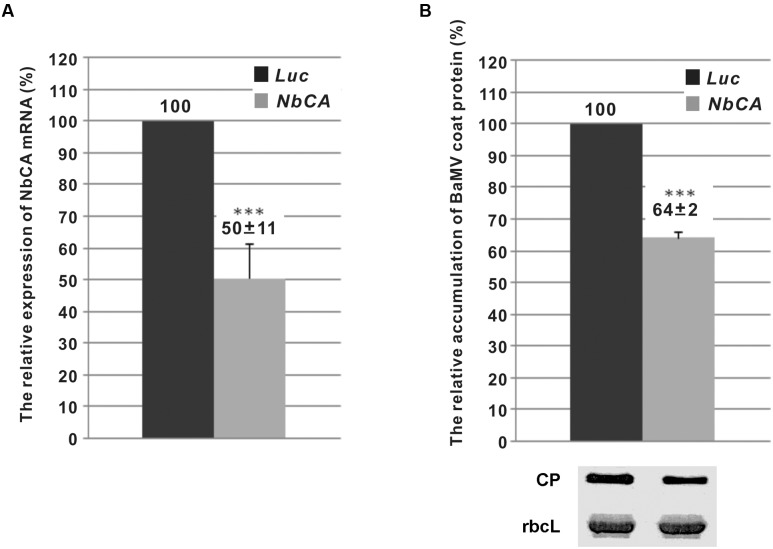
The relative expression of *NbCA* in *Nicotiana benthamiana* leaves with *NbCA-*knockdown and the accumulation of *Bamboo mosaic virus* (BaMV). **(A)** Real-time quantitative RT-PCR analysis of the efficiency of *NbCA* knockdown in *NbCA*- and *Luc*-knockdown leaves. The numbers above each bar are the mean relative expression of *NbCA* with the standard error obtained from at least three independent experiments. **(B)** Western blot analysis of the relative accumulation of BaMV coat protein in *Luc*- and *NbCA*-knockdown *N. benthamiana* leaves after 5 days post-inoculation (dpi). Total proteins were extracted from seven individual plants (*n* = 7). The numbers are the mean levels of coat protein with the standard error obtained from three independent experiments. The accumulation of BaMV coat protein in *Luc*-knockdown plants was set to 100%. *Luc*, *luciferase*-knockdown plants; *NbCA*, *NbCA*-knockdown plants; CP, coat protein; rbcL, Rubisco large subunit used as a loading control. ^∗∗∗^*p* < 0.001 by Student’s *t*-test.

The reduced *NbCA* expression in *N. benthamiana* did not cause any morphological change as compared with control plants (infiltrated with a TRV vector-carrying *luciferase* gene) (Supplementary Figure S1). The mRNA level of *NbCA* in *NbCA*-knockdown plants was approximately 50% that in the control plants (**Figure 1A**). At 5 days post-inoculation (dpi), the accumulation of BaMV in *NbCA*-knockdown plants was reduced to 64% that in *Luc*-knockdown control plants (**Figure 1B**). Hence, *NbCA* could be a positive regulator for BaMV infection in *N. benthamiana*.

### The Requirement of NbCA for Viral Replication Is Specific to BaMV

**FIGURE 2:**
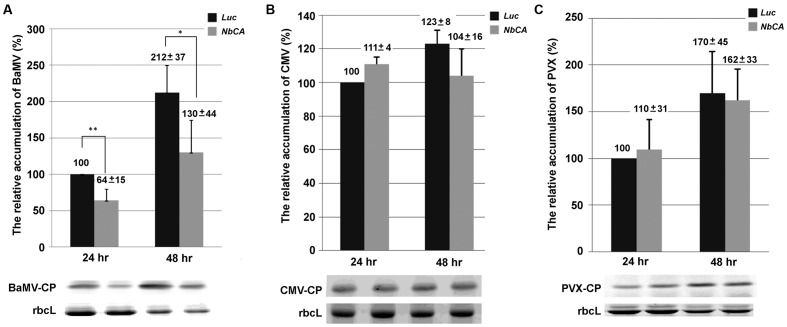
The relative accumulation of viral coat protein in *NbCA*-knockdown protoplasts. Western blot analysis of the accumulation of BaMV **(A)**, *Cucumber mosaic virus* (CMV) **(B)**, and *Potato virus X* (PVX) **(C)** coat protein extracted from *NbCA*- and *Luc*-knockdown protoplasts at 24 and 48 h post-inoculation (hpi). Protoplasts were isolated from *NbCA*- and *Luc*-knockdown *N. benthamiana* plants and inoculated with 1.5 μg BaMV, CMV, or PVX viral RNA. The accumulation of viral coat protein detected from *Luc*-knockdown protoplasts at 24 hpi was set to 100%. The numbers are the mean levels of coat protein with the standard error obtained from three independent experiments. *Luc*, luciferase-knockdown protoplasts; *NbCA*, *NbCA*-knockdown plants; CP, coat protein; rbcL, Rubisco large subunit used as a loading control; ^∗^*p* < 0.05, ^∗∗^*p* < 0.01 by Student’s *t*-test.

To determine whether the role of *NbCA* for BaMV accumulation is involved in virus replication or movement, cell wall-excluded protoplasts were prepared for viral RNA inoculation to eliminate the involvement of viral movement. The accumulation of BaMV coat protein in *NbCA-*knockdown protoplasts was reduced to 64 and 61% that of control protoplasts at 24 and 48 h post-inoculation (hpi), respectively (**Figure 2A**). To determine whether the involvement of *NbCA* is specific to the BaMV infection cycle, CMV and PVX were inoculated into *NbCA*-knockdown protoplasts. The accumulation of the coat protein of these two viruses in knockdown protoplasts did not differ from that in control protoplasts at 24 and 48 hpi (**Figures 2B,C**).

**FIGURE 3:**
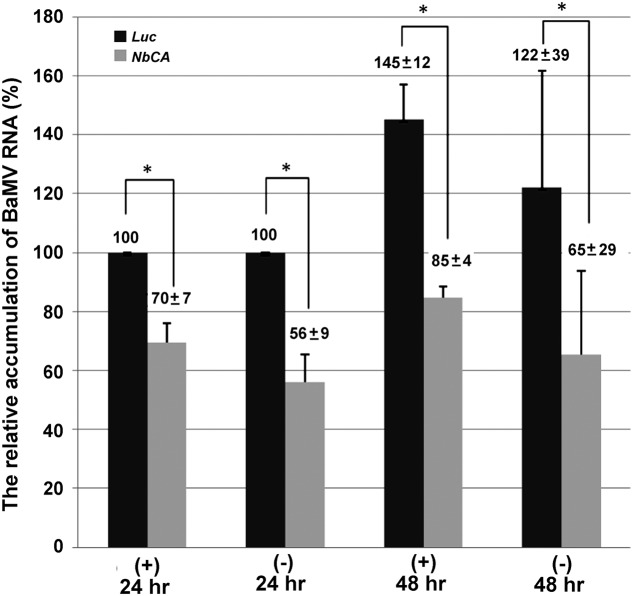
The relative accumulation of BaMV plus- and minus-strand RNA in *NbCA*-knockdown protoplasts. Real-time RT-PCR was used to quantify the accumulation of BaMV plus- and minus-strand RNA in *Luc*- and *NbCA*-knockdown *N. benthamiana* protoplasts at 24 and 48 hpi. The accumulation of BaMV RNA detected from *Luc*-knockdown protoplasts at 24 hpi was set to 100%. The numbers are the average accumulation of BaMV plus- and minus-strand RNA with the standard error obtained from three independent experiments; ^∗^*p* < 0.05 by Student’s *t*-test.

Furthermore, to elucidate whether this deficiency results from a defect in synthesizing the plus- or minus-strand viral RNA, qRT-PCR was used to quantify the accumulation of BaMV RNAs in knockdown protoplasts. At 24 hpi, the accumulation of the plus- and minus-strand of BaMV RNA in *NbCA*-knockdown protoplasts was reduced by approximately 30 and 44% as compared with control protoplasts. The similar reduced ratio in both plus- and minus-strand of BaMV RNA was observed at 48 hpi (42 and 47%, respectively). Therefore, the accumulation of plus- and minus-strand BaMV RNAs were similarly affected by the reduction in NbCA levels (**Figure 3**). These results suggest that NbCA is most likely involved in BaMV replication.

### NbCA Is Localized in *N. benthamiana* Chloroplasts

To clone the *NbCA* full-length gene, a primer was designed for the 3′ rapid amplification of cDNA ends (RACE) experiment to obtain the downstream sequence of ACAC10-1. The cDNA fragment derived from 3′ RACE contains the stop codon of *NbCA*. The upstream sequence of ACAC10-1 including the start codon of *NbCA* was retrieved from the transcriptome of the *N. benthamiana* draft genome (Hewett-Emmett and Tashian, 1996; Bombarely et al., 2012). Two specific primers were used to amplify the full-length *NbCA* coding region and cloned into the pEpyon binary vector (Chen et al., 2011), which carries the *mOrange2* reporter gene (OFP), to produce the NbCA-OFP fusion protein. Furthermore, the amino acid sequence of NbCA (accession no.: MF346699) was aligned with those from *N. tabacum* (NtCA; accession no.: P27141), and Arabidopsis (AtCA; accession no.: NP_186799) (Supplementary Figure S2). The sequence of NbCA shared 97 and 68% identity with those of NtCA and AtCA, respectively.

**FIGURE 4:**
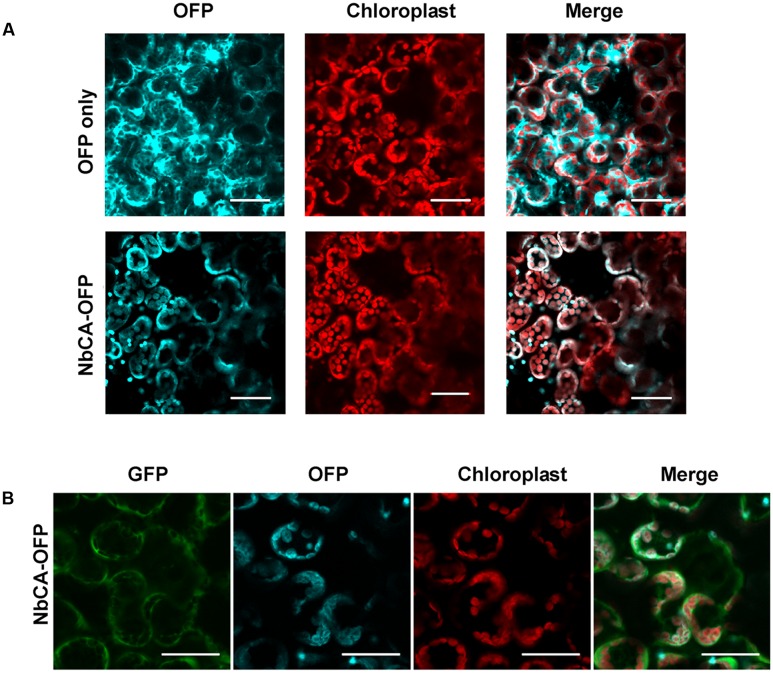
*Localization of NbCA in N. benthamiana cells.* The pEpyon vector and NbCA-Orange fluorescent protein (NbCA-OFP) constructs were transiently expressed on *N. benthamiana* leaves by agroinfiltration for 3 days without **(A)** or with **(B)** inoculation of BaMV vector carrying a GFP reporter. NbCA-OFP is labeled in cyan and chloroplast is in red. BaMV infection carrying GFP is in green. Images were taken under an Olympus Fluoview FV1000 Confocal Microscope with 488, 543, and 633 nm laser excitations for GFP, OFP, and autofluorescence, respectively. Scale bar: 40 μm.

To visualize the localization of NbCA in plant cells, NbCA-OFP was transiently expressed in *N. benthamiana* leaves by agroinfiltration to detect the fluorescent signal emitted from the OFP merged with the autofluorescence signal emitted from chloroplasts (**Figure 4**). NbCA was mainly localized in chloroplasts. Moreover, to observe whether the localization of NbCA was altered after BaMV inoculation, we co-infiltrated the infectious BaMV viral vector pKBG carrying green fluorescent protein (GFP) driven by subgenomic RNA promoter (Prasanth et al., 2011) with NbCA-OFP and found no re-localization of NbCA after BaMV inoculation (**Figure 4B**).

### NbCA Enhances BaMV Replication *in Vitro*

**FIGURE 5:**
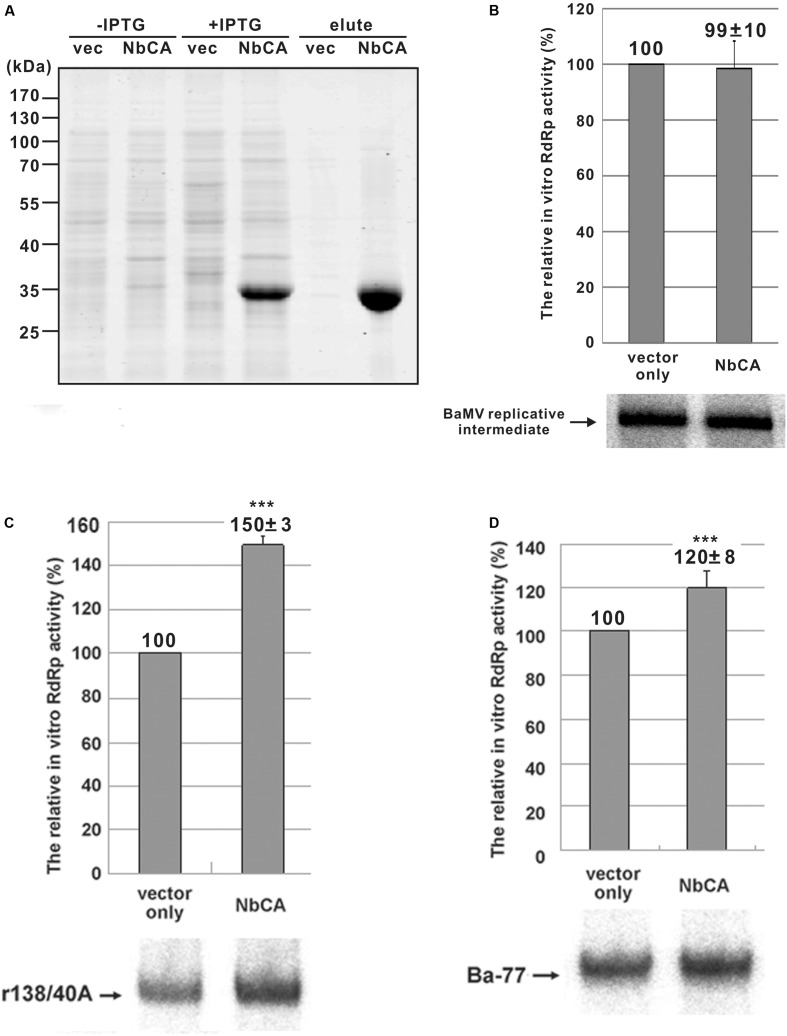
The expression of NbCA in *Escherichia coli* and *in vitro* replication assays with endogenous and exogenous templates. **(A)** Total proteins were extracted from *E. coli* that expressed vector only (vec) or NbCA with or without the induction of IPTG as indicated, separated on a 12% polyacrylamide gel/SDS, and stained with Coomassie blue. The eluents indicated as elute were the total proteins eluted from His-tag purification resin. *In vitro* replication assay involved use of the purified replicase complex from infected plants with the addition of *E. coli*-expressed proteins **(A)** to test the RdRp activity of the endogenous templates **(B)** and exogenous templates r138/40A **(C)** and Ba-77 **(D)**. RdRp activity with the addition of *E. coli*-expressed eluent of vector only was set to 100%. Representative results are shown. The numbers shown above each bar are the mean relative RdRp activity with the standard errors derived from at least three independent experiments. ^∗∗∗^*p* < 0.001 by Student’s *t*-test.

Since we found that the accumulation of BaMV coat protein and viral RNA was reduced in *NbCA*-knockdown plants (**Figure 1**) and protoplasts (**Figures 2**, **3**), NbCA may assist viral RNA replication. To validate this hypothesis, we transiently expressed NbCA-OFP in *N. benthamiana* followed by BaMV inoculation. However, accumulation of BaMV coat protein was not enhanced at 3 dpi. The pool of NbCA in cells may be enough for BaMV replication and the addition of exogenous NbCA by transient expression might not provide additional help for BaMV accumulation. Hence, we used *in vitro* replication (Cheng et al., 2001; Lin et al., 2005b) to exclude the effect of sufficient amount of CA in chloroplasts. We cloned and expressed the full-length CA in *E. coli* to acquire the purified-NbCA for *in vitro* replication experiments. The *E. coli* BL21 (DE3)-expressed recombinant NbCA-His was purified through a Nickel-chelating resin column (**Figure 5A**).

First, we tested whether NbCA affects endogenous RNA template activity, which represents the elongation step of BaMV replication. Viral RNA synthesis did not differ with or without the addition of the *E. coli*-expressed NbCA in the replication assay (**Figure 5B**). Second, we tested whether NbCA is involved in the initiation of BaMV replication. In the *in vitro* replication, we tested the two RNA templates, r138/40A (the 3′ UTR of BaMV, the promoter for minus-strand RNA synthesis) (Cheng et al., 2001) and Ba-77 (the 3′-end 77 nt of the minus-strand genome, the promoter for plus-strand RNA synthesis) (Lin et al., 2005a). The addition of NbCA in the *in vitro* replication assay with the exogenous templates r138/40A and Ba-77 increased RNA synthesis to 150 and 120%, respectively, that with vector alone (**Figures 5C,D**).

### BaMV Replication Could Be Regulated by the Proton Concentration

**FIGURE 6:**
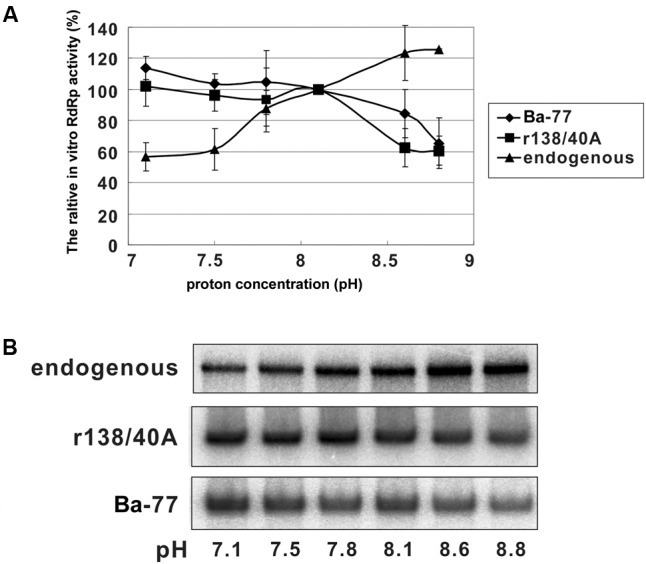
*In vitro* replication assay with the RNA templates in various pH conditions. **(A)** The relative RdRp activity of *in vitro* replication assay with endogenous and exogenous templates as indicated. The RdRp activity with pH 8.1 was set to 100%. Each point on the graph is the mean relative RdRp activity with the standard errors derived from three independent experiments. **(B)** Representative results of the *in vitro* replication assay.

Carbonic anhydrase activity condenses carbon dioxide with water to produce a free proton in the reaction. We wondered whether the proton concentration affects the viral RNA replication. In the *in vitro* replication assay, CA affected the exogenous but not the endogenous template activities. If the CA activity provides the free proton to change the micro-environment (reducing pH) such as the membrane-housed viral replication site, the condition for the re-initiation of the plus- or minus-strand RNA temples by BaMV replicase complex could be regulated. To test this hypothesis, we used various pH conditions for *in vitro* replication assays with endogenous and exogenous templates. The endogenous template (the viral RNAs already on the replicase complex and presumably at the elongation step) favored a higher pH condition (**Figure 6**). By contrast, the exogenous template (endogenous templates was removed by micrococcal nulcease and presumably at the re-initiation step) favored a lower pH condition. These results are implying that CA might be trapped into the viral replication site to produce free protons to create a more acidic microenvironment favoring the re-initiation of viral RNA replication.

## Discussion

In C4 plants, CA is mainly found in the cytoplasm and involved in converting CO_2_ into bicarbonate for carbon fixation (Hatch and Burnell, 1990). By contrast, β-CA activity is found mostly in the stroma of mesophyll chloroplasts in C3 plants (Poincelot, 1972), where it can represent up to 2% of total leaf protein (Okabe et al., 1984; Peltier et al., 2006). However, using antisense RNA to reduce this abundant chloroplast CA in C3 plants had only a marginal effect on CO_2_ assimilation as well as phenotypic changes (Price et al., 1994), which we observed (Supplementary Figure S1). Although the full-length CA of *N. benthamiana*, a C3 plant, has not yet been characterized, we obtained the coding region of *NbCA*, which showed 95% identity with *NtCA* (Supplementary Figure S2), and generated the NbCA-OFP to show chloroplast localization as predicted (**Figure 4**) (Fett and Coleman, 1994). Even though most research has revealed that CA suppression might reduce the HR response and thereby increase the susceptibility of pathogens (Slaymaker et al., 2002; Restrepo et al., 2005), CA positively regulated BaMV replication.

Carbonic anhydrase of alfalfa or tobacco was able to complement Δ*NCE103*, the *Saccharomyces cerevisiae* CA-like gene deletion strain sensitive to an oxidized environment such as in the presence of H_2_O_2_; hence, these two CAs were found to exhibit antioxidant activities (Gotz et al., 1999). Furthermore, NtCA exhibited enzymatic and antioxidant activities and also a salicylic acid-binding ability and was further called salicylic acid-binding protein 3 (SABP3) (Gotz et al., 1999; Slaymaker et al., 2002). One of the SABPs (designated SABP1) was identified as a cytosolic (peroxisomal) tobacco catalase, which exhibits H_2_O_2_-degrading activity (Chen et al., 1993a,b; Conrath et al., 1995). Accordingly, SABP3/NtCA or NbCA may also have antioxidant ability to degrade H_2_O_2_ and then dampen the load of host defense. Furthermore, one of the glutathione *S*-transferases (GSTs) was demonstrated to play an critical role in the minus-strand RNA synthesis of BaMV and was also involved in anti-oxidation processes in cells (Chen et al., 2013). Therefore, relieving oxidative stress by providing antioxidants such as GST or CA might provide an optimal condition for virus replication. In other words, disturbing the appropriate environment by reducing GST or CA could rapidly affect virus replication at the early time point of infection. We found reduced BaMV accumulation in the CA-knockdown *N. benthamiana* protoplasts at 24 hpi (**Figure 2A**). If NbCA is simply an antioxidant in general, it should favor both endogenous and exogenous templates in the *in vitro* replication assays. By contrast, the coat protein accumulation of CMV and another potexvirus, PVX, did not differ from that in the control (**Figure 2**). We assumed that chloroplast-localized NbCA would affect viruses that replicate in chloroplasts. CA is involved in various biological processes including SA binding (Slaymaker et al., 2002), however, the main receptors for SA signaling are unlikely in chloroplasts (Yan and Dong, 2014). Although SA is synthesized in chloroplast, it needs to be exported to the cytoplasm to regulate immune responses (Serrano et al., 2013). The SA-mediated defense pathway might be affected by virus infection (Li et al., 2016), but not simply affected by reducing CA expression.

Another possibility for NbCA assisting BaMV is fine-tuning the condition for viral RNA replication. Because BaMV replicates in chloroplasts and possibly associates with the thylakoid membrane in stroma (Cheng et al., 2013a). The pH value of the stroma is approximately 8, as the condition we have used in the *in vitro* replication assay (**Figure 6**). The replication complex associated with various host proteins including CA on the thylakoid membrane might create a replication competent microenvironment. Thus, the optimal condition for BaMV initiation and elongation could be regulated (**Figure 6**).

One of the CA activities in general is converting one carbon dioxide into bicarbonate and releasing one proton, which might act on the replicase complex and change the proton concentration at the microenvironment level to initiate RNA synthesis. Once the initiation kicks in, a switch from initiation to elongation is needed to increase pH for efficient elongation by turning off the NbCA activity or using another factor to replace NbCA. A possible candidate that could reduce the proton concentration is ferredoxin-NADP^+^ oxidoreductase (FNR). FNR transfers electrons from the reduced form of ferredoxin (Fd) to NADP+ and produces NADPH that consumes a proton with the reaction 2 Fd_red_ + NADP^+^ + H^+^ → 2 Fd_ox_ + NADPH (Mulo, 2011).

## Conclusion

We have identified a host factor that could assist in BaMV RNA replication. This factor, NbCA, could play a role in regulating the switch of initiation and elongation of RNA synthesis.

## Author Contributions

I-HC and C-HT designed the research, analyzed the data and wrote the manuscript. AT, Y-PH, I-FW, and S-FC performed the experiments. Y-HH and C-HT participated in data analysis and discussion.

## Conflict of Interest Statement

The authors declare that the research was conducted in the absence of any commercial or financial relationships that could be construed as a potential conflict of interest.

## References

[B1] Bombarely A. Rosli H. G. Vrebalov J. Moffett P. Mueller L. A. Martin G. B. (2012). A draft genome sequence of *Nicotiana benthamiana* to enhance molecular plant-microbe biology research. *Mol. Plant Microbe Interact.* 25 1523–1530. 10.1094/MPMI-06-12-0148-TA 22876960

[B2] Chen I. H. Chiu M. H. Cheng S. F. Hsu Y. H. Tsai C. H. (2013). The glutathione transferase of *Nicotiana benthamiana* NbGSTU4 plays a role in regulating the early replication of *Bamboo mosaic virus*. *New Phytol.* 199 749–757. 10.1111/nph.12304 23701112 PMC3744755

[B3] Chen I. H. Huang Y. W. Tsai C. H. (2017). The functional roles of the *cis*-acting elements in *Bamboo mosaic virus* RNA genome. *Front. Microbiol.* 8:645. 10.3389/fmicb.2017.00645 28450857 PMC5390519

[B4] Chen M. K. Hsu W. H. Lee P. F. Thiruvengadam M. Chen H. I. Yang C. H. (2011). The MADS box gene, FOREVER YOUNG FLOWER, acts as a repressor controlling floral organ senescence and abscission in Arabidopsis. *Plant J.* 68 168–185. 10.1111/j.1365-313X.2011.04677.x 21689171

[B5] Chen Z. Ricigliano J. W. Klessig D. F. (1993a). Purification and characterization of a soluble salicylic acid-binding protein from tobacco. *Proc. Natl. Acad. Sci. U.S.A.* 90 9533–9537. 8415736 10.1073/pnas.90.20.9533 PMC47603

[B6] Chen Z. Silva H. Klessig D. F. (1993b). Active oxygen species in the induction of plant systemic acquired resistance by salicylic acid. *Science* 262 1883–1886. 10.1126/science.8266079 8266079

[B7] Cheng C. W. Hsiao Y. Y. Wu H. C. Chuang C. M. Chen J. S. Tsai C. H. (2009). Suppression of *Bamboo mosaic virus* accumulation by a putative methyltransferase in *Nicotiana benthamiana*. *J. Virol.* 83 5796–5805. 10.1128/JVI.02471-08 19297487 PMC2681968

[B8] Cheng J. H. Ding M. P. Hsu Y. H. Tsai C. H. (2001). The partial purified RNA-dependent RNA polymerases from bamboo mosaic potexvirus and *Potato virus X* infected plants containing the template-dependent activities. *Virus Res.* 80 41–52. 10.1016/S0168-1702(01)00348-3 11597747

[B9] Cheng S. F. Huang Y. P. Chen L. H. Hsu Y. H. Tsai C. H. (2013a). Chloroplast phosphoglycerate kinase is involved in the targeting of *Bamboo mosaic virus* to chloroplasts in *Nicotiana benthamiana* plants. *Plant Physiol.* 163 1598–1608. 10.1104/pp.113.229666 24154620 PMC3846135

[B10] Cheng S. F. Tsai M. S. Huang C. L. Huang Y. P. Chen I. H. Lin N. S. (2013b). Ser/Thr kinase-like protein of *Nicotiana benthamiana* is involved in the cell-to-cell movement of *Bamboo mosaic virus*. *PLOS ONE* 8:e62907. 10.1371/journal.pone.0062907 23646157 PMC3639906

[B11] Cheng S. F. Huang Y. P. Wu Z. R. Hu C. C. Hsu Y. H. Tsai C. H. (2010). Identification of differentially expressed genes induced by *Bamboo mosaic virus* infection in *Nicotiana benthamiana* by cDNA-amplified fragment length polymorphism. *BMC Plant Biol.* 10:286. 10.1186/1471-2229-10-286 21184690 PMC3024324

[B12] Chou Y. L. Hung Y. J. Tseng Y. H. Hsu H. T. Yang J. Y. Wung C. H. (2013). The stable association of virion with the triple-gene-block protein 3-based complex of *Bamboo mosaic virus*. *PLOS Pathog.* 9:e1003405. 10.1371/journal.ppat.1003405 23754943 PMC3675025

[B13] Conrath U. Chen Z. Ricigliano J. R. Klessig D. F. (1995). Two inducers of plant defense responses, 2,6-dichloroisonicotinec acid and salicylic acid, inhibit catalase activity in tobacco. *Proc. Natl. Acad. Sci. U.S.A.* 92 7143–7147. 10.1073/pnas.92.16.7143 11607566 PMC41295

[B14] Dimario R. J. Clayton H. Mukherjee A. Ludwig M. Moroney J. V. (2017). Plant carbonic anhydrases: structures, locations, evolution, and physiological roles. *Mol. Plant* 10 30–46. 10.1016/j.molp.2016.09.001 27646307 PMC5226100

[B15] Fett J. P. Coleman J. R. (1994). Characterization and expression of two cDNAs encoding carbonic anhydrase in *Arabidopsis thaliana*. *Plant Physiol.* 105 707–713. 10.1104/pp.105.2.707 7520589 PMC159412

[B16] Floryszak-Wieczorek J. Arasimowicz-Jelonek M. (2017). The multifunctional face of plant carbonic anhydrase. *Plant Physiol. Biochem.* 112 362–368. 10.1016/j.plaphy.2017.01.007 28152407

[B17] Gotz R. Gnann A. Zimmermann F. K. (1999). Deletion of the carbonic anhydrase-like gene NCE103 of the yeast *Saccharomyces cerevisiae* causes an oxygen-sensitive growth defect. *Yeast* 15 855–864. 10.1002/(SICI)1097-0061(199907)15:10A<855::AID-YEA425>3.0.CO;2-C 10407265

[B18] Hatch M. D. Burnell J. N. (1990). Carbonic-anhydrase activity in leaves and its role in the first step of C-4 photosynthesis. *Plant Physiol.* 93 825–828. 10.1104/pp.93.2.825 16667544 PMC1062591

[B19] Hewett-Emmett D. Tashian R. E. (1996). Functional diversity, conservation, and convergence in the evolution of the alpha-, beta-, and gamma-carbonic anhydrase gene families. *Mol. Phylogenet. Evol.* 5 50–77. 10.1006/mpev.1996.0006 8673298

[B20] Hoang C. V. Chapman K. D. (2002). Biochemical and molecular inhibition of plastidial carbonic anhydrase reduces the incorporation of acetate into lipids in cotton embryos and tobacco cell suspensions and leaves. *Plant Physiol.* 128 1417–1427. 10.1104/pp.010879 11950990 PMC154269

[B21] Hsu Y. H. Wu C. W. Lin B. Y. Chen H. Y. Lee M. F. Tsai C. H. (1995). Complete genomic RNA sequences of *Cucumber mosaic virus* strain NT9 from Taiwan. *Arch. Virol.* 140 1841–1847. 10.1007/BF01384346 7503683

[B22] Hu H. Boisson-Dernier A. Israelsson-Nordstrom M. Bohmer M. Xue S. Ries A. (2010). Carbonic anhydrases are upstream regulators of CO2-controlled stomatal movements in guard cells. *Nat. Cell Biol.* 12 87–93. 10.1038/ncb2009 20010812 PMC2906259

[B23] Hu R. H. Lin M. C. Hsu Y. H. Meng M. (2011). Mutational effects of the consensus aromatic residues in the mRNA capping domain of *Bamboo mosaic virus* on GTP methylation and virus accumulation. *Virology* 411 15–24. 10.1016/j.virol.2010.12.022 21227477

[B24] Huang Y. L. Han Y. T. Chang Y. T. Hsu Y. H. Meng M. (2004). Critical residues for GTP methylation and formation of the covalent m7GMP-enzyme intermediate in the capping enzyme domain of *Bamboo mosaic virus*. *J. Virol.* 78 1271–1280. 10.1128/JVI.78.3.1271-1280.2004 14722282 PMC321370

[B25] Huang Y. P. Chen J. S. Hsu Y. H. Tsai C. H. (2013). A putative Rab-GTPase activation protein from *Nicotiana benthamiana* is important for *Bamboo mosaic virus* intercellular movement. *Virology* 447 292–299. 10.1016/j.virol.2013.09.021 24210126

[B26] Huang Y. P. Jhuo J. H. Tsai M. S. Tsai C. H. Chen H. C. Lin N. S. (2016). NbRABG3f, a member of Rab GTPase, is involved in *Bamboo mosaic virus* infection in *Nicotiana benthamiana*. *Mol. Plant Pathol.* 17 714–726. 10.1111/mpp.12325 26416342 PMC6638505

[B27] Huang Y. W. Hu C. C. Liou M. R. Chang B. Y. Tsai C. H. Meng M. H. (2012). Hsp90 interacts specifically with viral RNA and differentially regulates replication initiation of *Bamboo mosaic virus* and associated satellite RNA. *PLOS Pathog.* 8:e1002726. 10.1371/journal.ppat.1002726 22654666 PMC3359997

[B28] Hung C. J. Hu C. C. Lin N. S. Lee Y. C. Meng M. S. Tsai C. H. (2014a). Two key arginine residues in the coat protein of *Bamboo mosaic virus* differentially affect the accumulation of viral genomic and subgenomic RNAs. *Mol. Plant Pathol.* 15 196–210. 10.1111/mpp.12080 24393453 PMC6638855

[B29] Hung C. J. Huang Y. W. Liou M. R. Lee Y. C. Lin N. S. Meng M. H. (2014b). Phosphorylation of coat protein by protein kinase CK2 regulates cell-to-cell movement of *Bamboo mosaic virus* through modulating RNA binding. *Mol. Plant Microbe Interact.* 27 1211–1225. 10.1094/MPMI-04-14-0112-R 25025779

[B30] Lan P. Yeh W. B. Tsai C. W. Lin N. S. (2010). A unique glycine-rich motif at the N-terminal region of *Bamboo mosaic virus* coat protein is required for symptom expression. *Mol. Plant Microbe Interact.* 23 903–914. 10.1094/MPMI-23-7-0903 20521953

[B31] Lee C. C. Lin T. L. Lin J. W. Han Y. T. Huang Y. T. Hsu Y. H. (2015). Promotion of *Bamboo mosaic virus* accumulation in *Nicotiana benthamiana* by 5′→3′ exonuclease NbXRN4. *Front. Microbiol.* 6:1508. 10.3389/fmicb.2015.01508 26779163 PMC4702010

[B32] Li Y. Cui H. Cui X. Wang A. (2016). The altered photosynthetic machinery during compatible virus infection. *Curr. Opin. Virol.* 17 19–24. 10.1016/j.coviro.2015.11.002 26651024

[B33] Li Y. I. Chen Y. J. Hsu Y. H. Meng M. (2001a). Characterization of the AdoMet-dependent guanylyltransferase activity that is associated with the N terminus of *Bamboo mosaic virus* replicase. *J. Virol.* 75 782–788. 11134291 10.1128/JVI.75.2.782-788.2001 PMC113974

[B34] Li Y. I. Cheng Y. M. Huang Y. L. Tsai C. H. Hsu Y. H. Meng M. (1998). Identification and characterization of the *Escherichia coli*-expressed RNA-dependent RNA polymerase of *Bamboo mosaic virus*. *J. Virol.* 72 k10093–10099. 9811749 10.1128/jvi.72.12.10093-10099.1998 PMC110542

[B35] Li Y. I. Shih T. W. Hsu Y. H. Han Y. T. Huang Y. L. Meng M. (2001b). The helicase-like domain of plant potexvirus replicase participates in formation of RNA 5′ cap structure by exhibiting RNA 5′-triphosphatase activity. *J. Virol.* 75 12114–12120. 11711602 10.1128/JVI.75.24.12114-12120.2001 PMC116107

[B36] Lin J. W. Chiu H. N. Chen I. H. Chen T. C. Hsu Y. H. Tsai C. H. (2005a). Structural and functional analysis of the *cis*-acting elements required for plus-strand RNA synthesis of *Bamboo mosaic virus*. *J. Virol.* 79 k9046–9053. 15994798 10.1128/JVI.79.14.9046-9053.2005 PMC1168787

[B37] Lin J. W. Hsu Y. H. Tsai C. H. (2005b). Characterization of the infectivity of *Bamboo mosaic virus* with its correlation to the *in vitro* replicase activities in *Nicotiana benthamiana*. *Virus Res.* 112 77–84. 15904988 10.1016/j.virusres.2005.03.024

[B38] Lin J. W. Ding M. P. Hsu Y. H. Tsai C. H. (2007). Chloroplast phosphoglycerate kinase, a gluconeogenetic enzyme, is required for efficient accumulation of *Bamboo mosaic virus*. *Nucleic Acids Res.* 35 424–432. 10.1093/nar/gkl1061 17169994 PMC1802604

[B39] Lin M. K. Chang B. Y. Liao J. T. Lin N. S. Hsu Y. H. (2004). Arg-16 and Arg-21 in the N-terminal region of the triple-gene-block protein 1 of *Bamboo mosaic virus* are essential for virus movement. *J. Gen. Virol.* 85 251–259. 10.1099/vir.0.19442-0 14718640

[B40] Lin M. K. Hu C. C. Lin N. S. Chang B. Y. Hsu Y. H. (2006). Movement of potexviruses requires species-specific interactions among the cognate triple gene block proteins, as revealed by a trans-complementation assay based on the *Bamboo mosaic virus* satellite RNA-mediated expression system. *J. Gen. Virol.* 87 1357–1367. 10.1099/vir.0.81625-0 16603539

[B41] Lin N. S. Hsu Y. H. (1994). A satellite RNA associated with bamboo mosaic potexvirus. *Virology* 202 707–714. 10.1006/viro.1994.1392 7518162

[B42] Lin N. S. Lin B. Y. Lo N. W. Hu C. C. Chow T. Y. Hsu Y. H. (1994). Nucleotide sequence of the genomic RNA of bamboo mosaic potexvirus. *J. Gen. Virol.* 75 2513–2518. 10.1099/0022-1317-75-9-2513 8077956

[B43] Liu Y. Schiff M. Dinesh-Kumar S. P. (2002). Virus-induced gene silencing in tomato. *Plant J.* 31 777–786. 10.1046/j.1365-313X.2002.01394.x 12220268

[B44] Meng M. Lee C. C. (2017). Function and structural organization of the replication protein of *Bamboo mosaic virus*. *Front. Microbiol.* 8:522 10.3389/fmicb.2017.00522 PMC5368238 28400766

[B45] Moroney J. V. Bartlett S. G. Samuelsson G. (2001). Carbonic anhydrases in plants and algae. *Plant Cell Environ.* 24 141–153. 10.1111/j.1365-3040.2001.00669.x

[B46] Mulo P. (2011). Chloroplast-targeted ferredoxin-NADP(+) oxidoreductase (FNR): structure, function and location. *Biochim. Biophys. Acta* 1807 927–934. 10.1016/j.bbabio.2010.10.001 20934402

[B47] Okabe K. Yang S. Y. Tsuzuki M. Miyachi S. (1984). Carbonic-anhydrase - its content in spinach leaves and its taxonomic diversity studied with anti-spinach leaf carbonic-anhydrase antibody. *Plant Sci. Lett.* 33 145–153. 10.1016/0304-4211(84)90004-X

[B48] Peltier J. B. Cai Y. Sun Q. Zabrouskov V. Giacomelli L. Rudella A. (2006). The oligomeric stromal proteome of *Arabidopsis thaliana* chloroplasts. *Mol. Cell. Proteomics* 5 114–133. 10.1074/mcp.M500180-MCP200 16207701

[B49] Poincelot R. P. (1972). Intracellular distribution of carbonic anhydrase in spinach leaves. *Biochim. Biophys. Acta* 258 637–642. 10.1016/0005-2744(72)90255-0 4622001

[B50] Prasanth K. R. Huang Y. W. Liou M. R. Wang R. Y. Hu C. C. Tsai C. H. (2011). Glyceraldehyde 3-phosphate dehydrogenase negatively regulates the replication of *Bamboo mosaic virus* and its associated satellite RNA. *J. Virol.* 85 8829–8840. 10.1128/JVI.00556-11 21715476 PMC3165797

[B51] Price G. D. Voncaemmerer S. Evans J. R. Yu J. W. Lloyd J. Oja V. (1994). Specific reduction of chloroplast carbonic-anhydrase activity by antisense RNA in transgenic tobacco plants has a minor effect on photosynthetic CO2 assimilation. *Planta* 193 331–340. 10.1007/BF00201810

[B52] Restrepo S. Myers K. L. Del Pozo O. Martin G. B. Hart A. L. Buell C. R. (2005). Gene profiling of a compatible interaction between *Phytophthora infestans* and *Solanum tuberosum* suggests a role for carbonic anhydrase. *Mol. Plant Microbe Interact.* 18 913–922. 10.1094/MPMI-18-0913 16167762

[B53] Sawaya M. R. Cannon G. C. Heinhorst S. Tanaka S. Williams E. B. Yeates T. O. (2006). The structure of beta-carbonic anhydrase from the carboxysomal shell reveals a distinct subclass with one active site for the price of two. *J. Biol. Chem.* 281 7546–7555. 10.1074/jbc.M510464200 16407248

[B54] Serrano M. Wang B. Aryal B. Garcion C. Abou-Mansour E. Heck S. (2013). Export of salicylic acid from the chloroplast requires the multidrug and toxin extrusion-like transporter EDS5. *Plant Physiol.* 162 1815–1821. 10.1104/pp.113.218156 23757404 PMC3729763

[B55] Shaner N. C. Lin M. Z. Mckeown M. R. Steinbach P. A. Hazelwood K. L. Davidson M. W. (2008). Improving the photostability of bright monomeric orange and red fluorescent proteins. *Nat. Methods* 5 545–551. 10.1038/nmeth.1209 18454154 PMC2853173

[B56] Slaymaker D. H. Navarre D. A. Clark D. Del Pozo O. Martin G. B. Klessig D. F. (2002). The tobacco salicylic acid-binding protein 3 (SABP3) is the chloroplast carbonic anhydrase, which exhibits antioxidant activity and plays a role in the hypersensitive defense response. *Proc. Natl. Acad. Sci. U.S.A.* 99 11640–11645. 10.1073/pnas.182427699 12185253 PMC129322

[B57] So A. K. Espie G. S. Williams E. B. Shively J. M. Heinhorst S. Cannon G. C. (2004). A novel evolutionary lineage of carbonic anhydrase (epsilon class) is a component of the carboxysome shell. *J. Bacteriol.* 186 623–630. 10.1128/JB.186.3.623-630.2004 14729686 PMC321498

[B58] Tashian R. E. (1989). The carbonic anhydrases - widening perspectives on their evolution, expression and function. *Bioessays* 10 186–192. 10.1002/bies.950100603 2500929

[B59] Tripp B. C. Smith K. Ferry J. G. (2001). Carbonic anhydrase: new insights for an ancient enzyme. *J. Biol. Chem.* 276 48615–48618. 10.1074/jbc.R100045200 11696553

[B60] Tsai C. H. Cheng C. P. Peng C. W. Lin B. Y. Lin N. S. Hsu Y. H. (1999). Sufficient length of a poly(A) tail for the formation of a potential pseudoknot is required for efficient replication of bamboo mosaic potexvirus RNA. *J. Virol.* 73 2703–2709. 10074116 10.1128/jvi.73.4.2703-2709.1999 PMC104026

[B61] Yan S. Dong X. (2014). Perception of the plant immune signal salicylic acid. *Curr. Opin. Plant Biol.* 20 64–68. 10.1016/j.pbi.2014.04.006 24840293 PMC4143455

