# Supplementary material for: Nuclear-Encoded Plastidal Carbonic Anhydrase Is Involved in Replication of Bamboo mosaic virus RNA in Nicotiana benthamiana
Source: Front Microbiol. 2017 Oct 18;8:2046. doi: 10.3389/fmicb.2017.02046 (PMC5651272; doi:10.3389/fmicb.2017.02046)
Supplement: Supplementary file 1 [file Image_1.PDF]

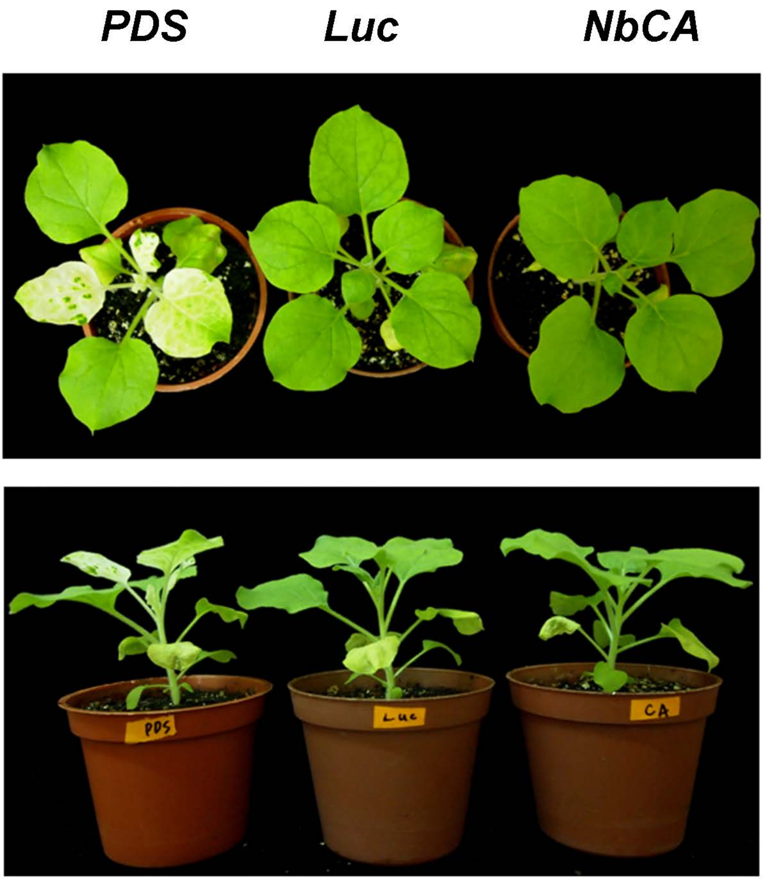

Supplemental Figure S1. *NbCA*-knockdown *Nicotiana benthamiana* showed no phenotype change. *Agrobacterium* containing pTRV2-phytoene desaturase (*PDS*), pTRV2-luciferase (*Luc*), pTRV2-*NbCA* was co-infiltrated with *Agrobacterium* containing pTRV1 onto 4-week-old *N. benthamiana*. Plants were monitored at 12 days post-infiltration. *PDS*-knockdown was a positive control and showed a photo-bleach phenotype. No significant differences between *NbCA*- and *Luc*-knockdown plants.

```
NtCA MSTASIN--SCLTISPAQASLKKPTR---------PVAFARLSNSSSSTS
NbCA MSTASIN--SCLTISPAQASLKKPTR---------PVAFARVSNSSSSPS
AtCA MSTAPLSGFFLTSLSPSQSSLQKLSLRTSSTVACLPPASSSSSSSSSSSS
     1........10........20........30........40.........

NtCA --VPSLIRNEPVFAAPTPIINPILREEMAKESYEQAIAALEKLLSEKGEL
NbCA --VPSLIRNEPVFAAPTPIINPILREEMANESYQQAIAALEKLLSEKGEL
AtCA RSVPTLIRNEPVFAAPAPIIAPYWSEEMGTEAYDEAIEALKKLLIEKEEL
     51.......60........70........80........90.........

NtCA GPIAAARVDQITAELQS--SDGSKPFDPVEHMKAGFIHFKTEKYEKNPAL
NbCA GPIAAARVDQITAELQS--SDGSKPFDPVEHMKAGFIHFKTEKYEKNPAL
AtCA KTVAAAKVEQITAALQTGTSSDKKAFDPVETIKQGFIKFKKEKYETNPAL
     101......110.......120.......130.......140........

NtCA YGELSKGQSPKFMVFACSDSRVCPSHVLNFQPGEAFVVRNIANMVPAYDK
NbCA YGELSKGQSPKYMVFACSDSRVCPSHILNFQPGEAFVVRNIANMVPAYDK
AtCA YGELAKGQSPKYMVFACSDSRVCPSHVLDFQPGDAFVVRNIANMVPPFDK
     151......160.......170.......180.......190........

NtCA TRYSGVGAAIEYAVLHLKVENIVVIGHSACGGIKGLMSLPADGSESTAFI
NbCA TRYSGVGAAIEYAVLHLKVENIIVIGHSACGGIKGLMSLPADGSESTAFI
AtCA VKYGGVGAAIEYAVLHLKVENIVVIGHSACGGIKGLMSFPLDGNNSTDFI
     201......210.......220.......230.......240........

NtCA EDWVKIGLPAKAKVQGEHVDKCFADQCTACEKEAVNVSLGNLLTYPFVRE
NbCA EDWVKIGLPAKAKVQGEHVDKCFADQCTACEEEAVNVSLGNLLTYPFVRE
AtCA EDWVKICLPAKSKVISELGDSAFEDQCGRCEREAVNVSLANLLTYPFVRE
     251......260.......270.......280.......290........

NtCA GLVKKTLALKGGHYDFVNGGFELWGLEFGLSPSLSV-----------
NbCA GLVKKTLALKGGHYDFVNGGFELWGLEFGLSPSLSV-----------
AtCA GLVKGTLALKGGYYDFVKGAFELWGLEFGLSETSSVKDVATILHWKL
     301......310.......320.......330.......340.....
```

Supplemental Figure S2. Amino acid sequence alignment of *N. benthamiana* carbonic anhydrase gene and its orthologs. Sequence alignment of amino acids of *N. tabacum* (NtCA; accession no.: P27141), Arabidopsis (AtCA; accession no.: NP_186799) and *N. benthamiana* CA gene (NbCA; accession no.: MF346699). Identical or similar residues are in yellow; different residues are in black.
